# Supplementary material for: Identification of Freezing-Responsive microRNAs and Their Targets in Chinese Jujube by Small RNA and Degradome Sequencing
Source: Int J Mol Sci. 2026 Sep 4;27(17):7912. doi: 10.3390/ijms27177912 (PMC13566890; doi:10.3390/ijms27177912)
Supplement: Supplementary file 1 [file ijms-27-07912-s001.zip › Supplementary Material S2/novel_miR_197.pdf]

Provisional ID	:	novel_miR_197
Score total	:	3.2
Score for star read(s)	:	-1.3
Score for read counts	:	0
Score for mfe	:	2.9
Score for randfold	:	1.6
Score for cons. seed	:	
Total read count	:	56
Mature read count	:	56
Loop read count	:	0
Star read count	:	0

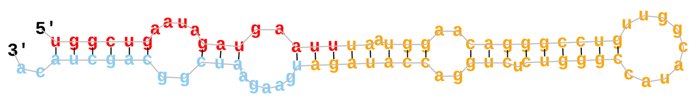

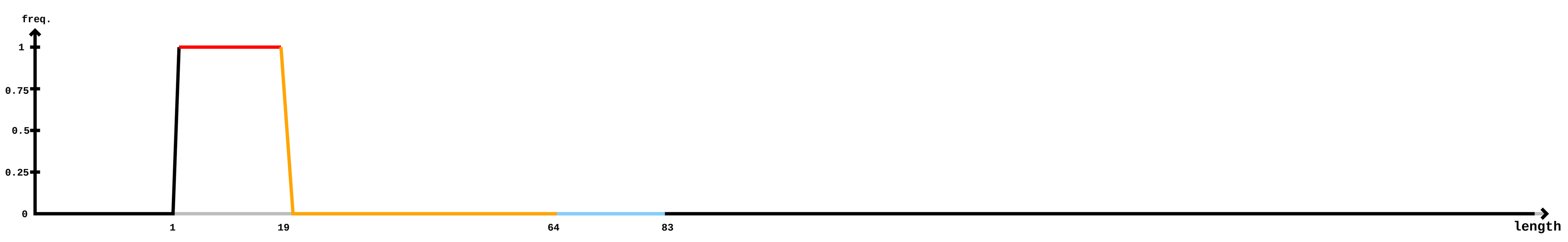

Mature **Star**

```
5'- auggauacgaggcgccuauuugugg cugaauagaugaauuuaauggaacagggccuguuggcauaccgggucucuggaccauagaugaagaaucggcagcuacagaugcuacugcacgauuagaagauauuugugcaggcugcaacauuccugggggggccaggugucccaggaguucuagguguuccaggagcaauaccaaaagguaggccagugggcacccaugaaguuguuggaaccaaguuaggagc   -3'   exp
    ..........((.((((((((((((((....(((..(((((.(((..(((((((((.........)))))).)))..)))))))))....)))..)))))))))))(((..((((((((.((.....)).))))))))...)))..(((((((((.((...)).))))))))))....(((((.(((...((..((((....)))).))...))))))))......(((.....))).....)))).))   reads   mm   sample
    .....................uggcugaauagauNaauu.......................................................................................................................................................................   1   1   LCK-1
    .....................ugAcugaauagaugaauu.......................................................................................................................................................................   1   1   LCK-2
    .....................uggcugaauagaugaauu.......................................................................................................................................................................   39   0   LCK-1
    .....................uggcugaauagaugaauu.......................................................................................................................................................................   15   0   LCK-2
```
